# Supplementary material for: Corticosteroids for severe acute exacerbations of chronic obstructive pulmonary disease in intensive care: From the French OUTCOMEREA cohort
Source: PLoS One. 2023 Apr 19;18(4):e0284591. doi: 10.1371/journal.pone.0284591 (PMC10115304; doi:10.1371/journal.pone.0284591)
Supplement: S1 Fig — Effects of corticosteroids on survival analysis (Cox model) for 28-day survival: HR = 0,89 [0.64; 1.24], p = 0.497. (DOCX) [file pone.0284591.s001.docx]

**S1 Fig. Survival curves at day 28 according to corticosteroid therapy for AECOPD at admission in ICU.** *Effects of corticosteroids on survival analysis (Cox model) for 28-day survival: HR=0,89 [0.64; 1.24], p=0.497*

**
