# Supplementary material for: Corticosteroids for severe acute exacerbations of chronic obstructive pulmonary disease in intensive care: From the French OUTCOMEREA cohort
Source: PLoS One. 2023 Apr 19;18(4):e0284591. doi: 10.1371/journal.pone.0284591 (PMC10115304; doi:10.1371/journal.pone.0284591)
Supplement: S2 Fig — Effects of corticosteroids in survival analysis (cox model) for 90-day survival: HR = 0.79 [0.6; 1.06], p = 0.121. (DOCX) [file pone.0284591.s002.docx]

**S2 Fig. Survival curves at day 90 according to corticosteroids therapy for AECOPD at admission in ICU (n=863).** *Effects of corticosteroids in survival analysis (cox model) for 90-day survival: HR=0.79 [0.6; 1.06], p=0.121*

**
