# Supplementary material for: Corticosteroids for severe acute exacerbations of chronic obstructive pulmonary disease in intensive care: From the French OUTCOMEREA cohort
Source: PLoS One. 2023 Apr 19;18(4):e0284591. doi: 10.1371/journal.pone.0284591 (PMC10115304; doi:10.1371/journal.pone.0284591)
Supplement: S3 Fig — Very severe COPD patients. Effects of corticosteroids in survival analysis (cox model) for 28-day survival for patients with a very severe COPD: HR = 1.21 [0.60; 2.42], p = 0.598. COPD: Chronic obstructive pulmonary disease. (DOCX) [file pone.0284591.s003.docx]

**S3 Fig. Survival curves at day 28 according to corticosteroids therapy for AECOPD at admission in ICU (n=265). Very severe COPD patients.** *Effects of corticosteroids in survival analysis (cox model) for 28-day survival for patients with a very severe COPD: HR=1.21 [0.60; 2.42], p=0.598. COPD: Chronic obstructive pulmonary disease.*

**
