# Supplementary material for: Corticosteroids for severe acute exacerbations of chronic obstructive pulmonary disease in intensive care: From the French OUTCOMEREA cohort
Source: PLoS One. 2023 Apr 19;18(4):e0284591. doi: 10.1371/journal.pone.0284591 (PMC10115304; doi:10.1371/journal.pone.0284591)
Supplement: S4 Fig — Not very Severe COPD patients or unknown COPD severity. Effects of corticosteroids in survival analysis (cox model) for 28-day survival for patients with not very severe COPD or unknown COPD Severity: HR = 0.85 [0.58; 1.26], p = 0.420. COPD: Chronic obstructive pulmonary disease. (DOCX) [file pone.0284591.s004.docx]

**S4 Fig. Survival curves at day 28 according to corticosteroids therapy for AECOPD at admission in ICU (n=982). Not very Severe COPD patients or unknown COPD severity.** *Effects of corticosteroids in survival analysis (cox model) for 28-day survival for patients with not very severe COPD or unknown COPD Severity: HR=0.85 [0.58; 1.26], p=0.420.*

*COPD: Chronic obstructive pulmonary disease.*

**
