# Supplementary material for: Corticosteroids for severe acute exacerbations of chronic obstructive pulmonary disease in intensive care: From the French OUTCOMEREA cohort
Source: PLoS One. 2023 Apr 19;18(4):e0284591. doi: 10.1371/journal.pone.0284591 (PMC10115304; doi:10.1371/journal.pone.0284591)
Supplement: S5 Fig — All patients and sub-groups of patients. VFD at day 28 is express in Median and interquartiles of days, Median [Q1; Q3]. IRR: Incidence Rate Ratio. VFD: Ventilator-free days. IMV: Invasive Mechanical Ventilation. NIV: Non-Invasive Ventilation. (DOCX) [file pone.0284591.s005.docx]

**S5 Fig. Summary of results for corticosteroids therapy and ventilator-free days. All patients and sub-groups of patients.** *VFD at day 28 is express in Median and interquartiles of days, Median [Q1; Q3]. IRR: Incidence Rate Ratio. VFD: ventilator-free days. IMV: Invasive Mechanical Ventilation. NIV: Non-Invasive Ventilation.*

**
