# Supplementary material for: Corticosteroids for severe acute exacerbations of chronic obstructive pulmonary disease in intensive care: From the French OUTCOMEREA cohort
Source: PLoS One. 2023 Apr 19;18(4):e0284591. doi: 10.1371/journal.pone.0284591 (PMC10115304; doi:10.1371/journal.pone.0284591)
Supplement: S6 Fig — ICU: Intensive Care Unit. (DOCX) [file pone.0284591.s006.docx]

**S6 Fig. Summary of results for corticosteroids therapy and antibiotic consumption.** *ICU: Intensive Care Unit*
