# Supplementary material for: Corticosteroids for severe acute exacerbations of chronic obstructive pulmonary disease in intensive care: From the French OUTCOMEREA cohort
Source: PLoS One. 2023 Apr 19;18(4):e0284591. doi: 10.1371/journal.pone.0284591 (PMC10115304; doi:10.1371/journal.pone.0284591)
Supplement: S7 Fig — AFD is express in Median and interquartiles of days, Median [Q1; Q3]. IRR: Incidence Rate Ratio. AFD: Antibiotic-free days. LOS: Length of stay. (DOCX) [file pone.0284591.s007.docx]

**S7 Fig. Summary of results for corticosteroids therapy regarding the endpoint “Alive and antibiotic-free days” at 5 days, 10 days and 15 days.** *AFD is express in Median and interquartiles of days, Median [Q1; Q3] IRR: Incidence Rate Ratio. AFD: Antibiotic-free days. LOS: Length of stay*

**
