# Supplementary material for: Corticosteroids for severe acute exacerbations of chronic obstructive pulmonary disease in intensive care: From the French OUTCOMEREA cohort
Source: PLoS One. 2023 Apr 19;18(4):e0284591. doi: 10.1371/journal.pone.0284591 (PMC10115304; doi:10.1371/journal.pone.0284591)
Supplement: S1 Table — ICU: Intensive Care Unit. NIV: Non-Invasive Ventilation. IMV: Invasive Mechanical Ventilation. (DOCX) [file pone.0284591.s008.docx]

**S1 Table. Ventilatory support used for patients admitted in ICU for a severe AECOPD.** *ICU: Intensive Care Unit. NIV: Non-Invasive Ventilation. IMV: Invasive Mechanical Ventilation*

|  | Median [Q1; Q3] or number (Percentage) |
| --- | --- |
| **Type of Ventilatory Support** | |
| Necessity of ventilatory support, n(%) | 1034 (82.9) |
| Necessity of IMV, n(%) | 540 (43.3) |
| Only NIV as ventilatory support, n(%) | 494 (39.6) |
| **Ventilatory support at ICU admission** | |
| Necessity of ventilatory support at admission in ICU, n(%) | 990 (79.4) |
| IMV at admission in ICU, n(%) | 455 (36.5) |
| Only NIV at admission in ICU, n(%) | 535 (42.9) |
| **Duration of mechanical ventilation** | |
| ***Patients with NIV as ventilatory support*** | |
| Duration of use of NIV (days) (n=494) | 3.0 [2.0; 5.0] |
| ***Patients with IMV as ventilatory support*** | |
| Duration of use of IMV (days) (n=540) | 8.0 [4.0; 16.0] |
