# Supplementary material for: Corticosteroids for severe acute exacerbations of chronic obstructive pulmonary disease in intensive care: From the French OUTCOMEREA cohort
Source: PLoS One. 2023 Apr 19;18(4):e0284591. doi: 10.1371/journal.pone.0284591 (PMC10115304; doi:10.1371/journal.pone.0284591)
Supplement: S2 Table — Statistical analysis performed with khi2 Test and Wilcoxon-Mann-Whitney Test. ICU: Intensive Care Unit. AECOPD: Acute exacerbation of chronic obstructive pulmonary disease. BMI: Body Mass Index. SOFA: Sequential Organ Failure Assessment. NIV: Non-Invasive Ventilation. IMV: Invasive Mechanical Ventilation. Pa02: Partial pressure of oxygen. FiO2: Fraction of inspired oxygen. PaCO2: Partial Pressure of Carbon Dioxide. (DOCX) [file pone.0284591.s009.docx]

**S2 Table. Characteristics of patients regarding the prescription of corticosteroids at ICU admission.** *Statistical analysis performed with khi2 Test and Wilcoxon-Mann-Whitney Test*

*ICU: Intensive Care Unit. AECOPD: Acute exacerbation of chronic obstructive pulmonary disease. BMI: Body Mass Index. SOFA: Sequential Organ Failure Assessment. NIV: Non-Invasive Ventilation. IMV: Invasive Mechanical Ventilation. Pa02: Partial pressure of oxygen. FiO2: Fraction of inspired oxygen. PaCO2: Partial Pressure of Carbon Dioxide.*

|  | **No Corticosteroids at admission in ICU (n=856)** | **Corticosteroids at admission in ICU (n=391)** | **p-value** |
| --- | --- | --- | --- |
| **Variables** | Median [Q1; Q3] or number (Percentage) | |  |
|  | | | |
| **Characteristics and severity at ICU admission** | | | |
| Age (years) | 71 [62; 78] | 70 [61; 77] | 0.424 |
| Male gender, n(%) | 563 (65.8) | 240 (61.4) | 0.133 |
| BMI (n=968), n(%) | 25.2 [20.9; 31.1] | 24.3 [21.1; 29.4] | 0.212 |
| < 18.5 | 81 (12.3) | 42 (13.5) | 0.162 |
| 18.5 - 24.9 | 242 (36.8) | 128 (41.2) |  |
| 25 - 29.9 | 143 (21.8) | 77 (24.8) |  |
| 30 - 34.9 | 97 (14.8) | 32 (10.3) |  |
| 35 - 39.9 | 48 (7.3) | 16 (5.1) |  |
| ≥ 40 | 46 (7.0) | 16 (5.1) |  |
| SAPS 2 Score, n(%) | 37 [29; 48] | 37 [28; 47] | 0.447 |
| SOFA Day-1 | 4 [2; 6] | 3 [2; 6] | 0.028 |
| < 3, n(%) | 245 (28.6) | 143 (36.6) | 0.035 |
| 3 - 5, n(%) | 337 (39.4) | 144 (36.8) |  |
| 6 - 8, n(%) | 174 (20.3) | 68 (17.4) |  |
| ≥ 9, n(%) | 100 (11.7) | 36 (9.2) |  |
| PaO_2_/FiO_2_ ratio at admission (mmHg) (n=1173) | 200 [133; 281] | 219 [150; 298] | 0.006 |
| < 100, n(%) | 118 (14.9) | 41 (10.8) | 0.070 |
| 100 - 199, n(%) | 278 (35.0) | 119 (31.4) |  |
| 200 - 299, n(%) | 222 (28.0) | 125 (33.0) |  |
| ≥ 300, n(%) | 176 (22.2) | 94 (24.8) |  |
| Limitation of therapeutic effort during ICU stay, n(%) | 147 (17.2) | 60 (15.4) | 0.421 |
| Limitation of therapeutic effort at admission in ICU, n(%) | 64 (7.5) | 32 (8.2) | 0.664 |
|  | | | |
| **COPD Severity** | | | |
| Very Severe COPD (Oxygen therapy at home or NIV at home or Stage 4 Airflow limitation) | 134 (15.7) | 131 (33.50) | <.001 |
| Non-Very Severe COPD | 319 (37.3) | 127 (32.48) |  |
| Unknown COPD Severity | 403 (47.1) | 133 (34.02) |  |
|  | | | |
| **Ventilatory support** | | | |
| *Ventilatory support during ICU Stay* | | | |
| Necessity of ventilatory support, n(%) | 706 (82.5) | 328 (83.9) | 0.539 |
| Necessity of IMV, n(%) | 372 (43.5) | 168 (43.0) | 0.871 |
| NIV as unique ventilatory support, n(%) | 334 (39.0) | 160 (40.9) | 0.524 |
| *Ventilatory support at admission in ICU* | | | |
| Necessity of ventilatory support at admission in ICU, n(%) | 669 (78.2) | 321 (82.1) | 0.110 |
| IMV at admission in ICU, n(%) | 308 (36.0) | 147 (37.6) | 0.583 |
| Only NIV at admission in ICU, n(%) | 361 (42.2) | 174 (44.5) | 0.441 |
| *Patients with NIV as unique ventilatory support* | | | |
| Length of use of NIV (days) (n=494) | 3 [2; 5] | 3 [2; 5] | 0.829 |
| *Patients with IMV as ventilatory support* | | | |
| Length of use of IMV (days) (n=540) | 8 [4; 17] | 9 [4; 16] | 0.597 |
|  | | | |
| NIV failure among patients with NIV as first ventilatory support (n=625), n(%) | 96 (23.1) | 56 (26.7) | 0.331 |
|  | | | |
| **Comorbid condition** | | | |
| Arterial hypertension, n(%) | 224 (26.2) | 85 (21.74) | 0.093 |
| Heart disease, n(%) | 165 (19.3) | 90 (23.02) | 0.128 |
| Diabetes mellitus, n(%) | 171 (20.0) | 64 (16.37) | 0.131 |
| Obesity (n=1014), n(%) | 229 (33.0) | 72 (22.57) | <.001 |
|  | | | |
| **Cause of acute exacerbation of COPD** | | | |
| Respiratory infection, n(%) | 592 (69.2) | 301 (77.0) | 0.004 |
| Respiratory except respiratory infection, n(%) | 162 (18.9) | 64 (16.4) | 0.277 |
| Cardiac except pulmonary oedema, n(%) | 29 (3.4) | 9 (2.3) | 0.301 |
| Postoperative, n(%) | 9 (1.1) | 1 (0.3) | 0.144 |
| Neurologic, n(%) | 17 (2.1) | 2 (0.5) | 0.049 |
| Digestive, n(%) | 11 (1.3) | 4 (1.0) | 0.694 |
| Other, n(%) | 26 (3.0) | 6 (1.5) | 0.119 |
|  | | | |
| **Timing to ICU admission** | | | |
| Direct ICU admission or < 24h after hospital admission | 704 (82.2) | 326 ( 83.4) | 0.248 |
| ICU admission > 24h and ≤ 7 days after hospital admission | 86 (10.0) | 29 (7.4) |  |
| ICU admission > 7 days after hospital admission | 66 (7.7) | 36 (9.2) |  |
|  | | | |
| **Arterial blood gases at admission** | | | |
| pH (mmHg) (n=1108) | 7.34 [7.26; 7.41] | 7.31 [7.23; 7.38] | <.001 |
| < 7.25 | 165 (22.0) | 103 (28.8) | <.001 |
| 7.25 - 7.29 | 106 (14.1) | 56 (15.6) |  |
| 7.30 - 7.34 | 132 (17.6) | 80 (22.4) |  |
| ≥ 7.35 | 347 (46.3) | 119 (33.2) |  |
| PaO2 (mmHg) (n=1199), n(%) | 74 [60; 101] | 81 [65; 112] | <.001 |
| PaCO2 (mmHg) (1200), n(%) | 57 [44; 73] | 62 [47; 77] | 0.006 |
| HCO3- (mmHg) (n=1124), n(%) | 29.0 [24.5; 34.0] | 29.0 [25.0; 34.0] | 0.360 |
