# Supplementary material for: Corticosteroids for severe acute exacerbations of chronic obstructive pulmonary disease in intensive care: From the French OUTCOMEREA cohort
Source: PLoS One. 2023 Apr 19;18(4):e0284591. doi: 10.1371/journal.pone.0284591 (PMC10115304; doi:10.1371/journal.pone.0284591)
Supplement: S3 Table — Adjustment also performed on centre. ICU: Intensive Care Unit. AECOPD: Acute exacerbation of chronic obstructive pulmonary disease. IPTW: Inverse Probability of Treatment Weighting. BMI: Body Mass Index. SOFA: Sequential Organ Failure Assessment. Pa02: Partial pressure of oxygen. FiO2: Fraction of inspired oxygen. NIV: Non-Invasive Ventilation. IMV: Invasive Mechanical Ventilation. (DOCX) [file pone.0284591.s010.docx]

**S3 Table. Weight model used to compute IPTW - Corticosteroids treatment for AECOPD at admission in ICU.** *Adjustment also performed on centre and year. ICU: Intensive Care Unit. AECOPD: Acute exacerbation of chronic obstructive pulmonary disease. IPTW: Inverse Probability of Treatment Weighting. BMI: Body Mass Index. SOFA: Sequential Organ Failure Assessment. Pa02: Partial pressure of oxygen. FiO2: Fraction of inspired oxygen. NIV: Non-Invasive Ventilation. IMV: Invasive Mechanical Ventilation.*

| **Variables used to compute IPTW** | **Odds Ratio [95%CI]** | **p-value** |
| --- | --- | --- |
| **Characteristics of patients** | | |
| Age | 1.00 [0.98; 1.01] | 0.484 |
| Male gender | 0.80 [0.61; 1.05] | 0.104 |
| BMI | 0.62 [0.38; 0.99] | 0.046 |
|  | | |
| **Characteristics of AECOPD at ICU admission** | | |
| SOFA Day-1 | 0.97 [0.92; 1.02] | 0.284 |
| PaO_2_/FiO_2_ ratio | 1.40 [1.09; 1.81] | 0.009 |
| pH | 0.25 [0.07; 0.84] | 0.025 |
| Only NIV | 1.02 [0.71; 1.46] | 0.925 |
| IMV | 1.24 [0.83; 1.85] | 0.297 |
| Limitation of therapeutic effort | 0.84 [0.51; 1.38] | 0.495 |
| Respiratory infection as cause of AECOPD | 1.52 [1.13; 2.03] | 0.005 |
|  | | |
| **Timing to ICU admission** | | |
| ICU admission > 24h and ≤ 7 days after hospital admission | 0.81 [0.51; 1.30] | 0.489 |
| ICU admission > 7 days after hospital admission | 1.18 [0.75; 1.87] |  |
| Direct ICU admission or < 24h after hospital admission | 1 [.; .] |  |
|  | | |
| **Characteristics of COPD disease** | | |
| Very Severe COPD | 2.20 [1.57; 3.06] | <.001 |
| COPD Status unknown | 0.99 [0.65; 1.51] |  |
| No Very Severe COPD | 1 [.; .] |  |
