# Supplementary material for: Corticosteroids for severe acute exacerbations of chronic obstructive pulmonary disease in intensive care: From the French OUTCOMEREA cohort
Source: PLoS One. 2023 Apr 19;18(4):e0284591. doi: 10.1371/journal.pone.0284591 (PMC10115304; doi:10.1371/journal.pone.0284591)
Supplement: S4 Table — Adjustment also performed on centre and year. ICU: Intensive Care Unit. AECOPD: Acute exacerbation of chronic obstructive pulmonary disease. IPTW: Inverse Probability of Treatment Weighting. BMI: Body Mass Index. SOFA: Sequential Organ Failure Assessment. Pa02: Partial pressure of oxygen. FiO2: Fraction of inspired oxygen. NIV: Non-Invasive Ventilation. IMV: Invasive Mechanical Ventilation. (DOCX) [file pone.0284591.s011.docx]

**S4 Table. Double Robust Analysis of the association between prescription of corticosteroids at admission in ICU for AECOPD and primary composite outcome: death or invasive mechanical ventilation at Day 28.** *Adjustment also performed on centre and year.*

*ICU: Intensive Care Unit. AECOPD: Acute exacerbation of chronic obstructive pulmonary disease. IPTW: Inverse Probability of Treatment Weighting. BMI: Body Mass Index. SOFA: Sequential Organ Failure Assessment. Pa02: Partial pressure of oxygen. FiO2: Fraction of inspired oxygen. NIV: Non-Invasive Ventilation. IMV: Invasive Mechanical Ventilation.*

| **Variables** | **Odds Ratio [95%CI]** | **p-value** |
| --- | --- | --- |
| **Corticosteroids Therapy** | | |
| Corticosteroids at ICU admission | 0.70 [0.49; 0.99] | 0.044 |
| **Characteristics of patients** | | |
| Age | 1.05 [1.03; 1.06] | <.001 |
| Male gender | 1.29 [0.92; 1.80] | 0.138 |
| BMI | 0.93 [0.50; 1.71] | 0.813 |
|  | | |
| **Characteristics of AECOPD at ICU admission** | | |
| SOFA Day-1 | 1.17 [1.10; 1.24] | <.001 |
| PaO_2_/FiO_2_ ratio | 1.23 [0.89; 1.70] | 0.200 |
| pH | 0.88 [0.20; 3.80] | 0.867 |
| Only NIV | 1.21 [0.71; 2.06] | 0.475 |
| IMV | 2.52 [1.47; 4.31] | <.001 |
| Limitation of therapeutic effort | 3.41 [2.09; 5.58] | <.001 |
| Respiratory infection as cause of AECOPD | 0.78 [0.56; 1.10] | 0.160 |
|  | | |
| **Timing to ICU admission** | | |
| ICU admission > 24h and ≤ 7 days after hospital admission | 1.77 [1.06; 2.94] | 0.005 |
| ICU admission > 7 days after hospital admission | 2.03 [1.24; 3.33] |  |
| Direct ICU admission or < 24h after hospital admission | 1 [.; .] |  |
|  | | |
| **Characteristics of COPD disease** | | |
| Very severe COPD | 0.89 [0.57; 1.40] | 0.552 |
| COPD severity unknown | 1.23 [0.73; 2.07] |  |
| No very severe COPD | 1 [.; .] |  |
