# Supplementary material for: Corticosteroids for severe acute exacerbations of chronic obstructive pulmonary disease in intensive care: From the French OUTCOMEREA cohort
Source: PLoS One. 2023 Apr 19;18(4):e0284591. doi: 10.1371/journal.pone.0284591 (PMC10115304; doi:10.1371/journal.pone.0284591)
Supplement: S5 Table — Adjustment also performed on centre and year. Survival analysis performed with a cox model. ICU: Intensive Care Unit. AECOPD: Acute exacerbation of chronic obstructive pulmonary disease. COPD: Chronic Obstructive Pulmonary Disease. BMI: Body Mass Index. SOFA: Sequential Organ Failure Assessment. Pa02: Partial pressure of oxygen. FiO2: Fraction of inspired oxygen. NIV: Non-Invasive Ventilation. IMV: Invasive Mechanical Ventilation. (DOCX) [file pone.0284591.s012.docx]

**S5 Table. Double Robust Analysis of the association between prescription of corticosteroids at admission in ICU for AECOPD and in-ICU death.** *Adjustment also performed on centre and year. Survival analysis performed with a cox model. ICU: Intensive Care Unit. AECOPD: Acute exacerbation of chronic obstructive pulmonary disease. COPD: Chronic Obstructive Pulmonary Disease. BMI: Body Mass Index. SOFA: Sequential Organ Failure Assessment. Pa02: Partial pressure of oxygen. FiO2: Fraction of inspired oxygen. NIV: Non-Invasive Ventilation. IMV: Invasive Mechanical Ventilation.*

| **Variables** | **Hazard Ratio [95%CI]** | **p-value** |
| --- | --- | --- |
| **Corticosteroids Therapy** | | |
| Corticosteroids at ICU admission | 0.81 [0.56; 1.17] | 0.263 |
|  | | |
| **Characteristics of patients** | | |
| Age | 1.04 [1.02; 1.06] | <.001 |
| Male gender | 1.26 [0.89; 1.79] | 0.197 |
| BMI | 0.86 [0.45; 1.64] | 0.648 |
|  | | |
| **Characteristics of AECOPD at ICU admission** | | |
| SOFA Day-1 | 1.15 [1.08; 1.21] | <.001 |
| PaO_2_/FiO_2_ ratio | 1.15 [0.83; 1.59] | 0.40 |
| pH | 0.33 [0.08; 1.37] | 0.127 |
| Only NIV | 1.44 [0.73; 2.72] | 0.281 |
| IMV | 2.30 [1.19; 4.43] | 0.013 |
| Limitation of therapeutic effort | 3.1 [2.03; 4.73] | <.001 |
| Respiratory infection as cause of AECOPD | 0.79 [0.56; 1.12] | 0.188 |
|  | | |
| **Timing to ICU admission** | | |
| ICU admission > 24h and ≤ 7 days after hospital admission | 1.03 [0.56; 1.87] | 0.897 |
| ICU admission > 7 days after hospital admission | 1.12 [0.68; 1.86] |  |
| Direct ICU admission or < 24h after hospital admission | 1 [.; .] |  |
|  | | |
| **Characteristics of COPD disease** | | |
| Very severe COPD | 1.05 [0.67; 1.67] | 0.680 |
| COPD severity unknown | 1.28 [0.73; 2.25] |  |
| No very severe COPD | 1 [.; .] |  |
