# Supplementary material for: Corticosteroids for severe acute exacerbations of chronic obstructive pulmonary disease in intensive care: From the French OUTCOMEREA cohort
Source: PLoS One. 2023 Apr 19;18(4):e0284591. doi: 10.1371/journal.pone.0284591 (PMC10115304; doi:10.1371/journal.pone.0284591)
Supplement: S6 Table — Adjustment also performed on centre and year. Survival analysis performed with a cox model. ICU: Intensive Care Unit. AECOPD: Acute exacerbation of chronic obstructive pulmonary disease. COPD: Chronic Obstructive Pulmonary Disease. BMI: Body Mass Index. SOFA: Sequential Organ Failure Assessment. Pa02: Partial pressure of oxygen. FiO2: Fraction of inspired oxygen. NIV: Non-Invasive Ventilation. IMV: Invasive Mechanical Ventilation. (DOCX) [file pone.0284591.s013.docx]

**S6 Table. Double Robust Analysis of the association between prescription of corticosteroids at admission in ICU for AECOPD and in-hospital death.** *Adjustment also performed on centre and year. Survival analysis performed with a cox model. ICU: Intensive Care Unit. AECOPD: Acute exacerbation of chronic obstructive pulmonary disease. COPD: Chronic Obstructive Pulmonary Disease. BMI: Body Mass Index. SOFA: Sequential Organ Failure Assessment. Pa02: Partial pressure of oxygen. FiO2: Fraction of inspired oxygen. NIV: Non-Invasive Ventilation. IMV: Invasive Mechanical Ventilation.*

| **Variables used to compute IPTW** | **Hazard Ratio [95%CI]** | **p-value** |
| --- | --- | --- |
| **Corticosteroids Therapy** | | |
| Corticosteroids at ICU admission | 0.77 [0.57; 1.04] | 0.085 |
|  | | |
| **Characteristics of patients** | | |
| Age | 1.05 [1.03; 1.06] | <.001 |
| Male gender | 1.17 [0.89; 1.54] | 0.268 |
| BMI | 0.4 [0.39; 1.08] | 0.093 |
|  | | |
| **Characteristics of AECOPD at ICU admission** | | |
| SOFA Day-1 | 1.14 [1.08; 1.19] | <.001 |
| PaO_2_/FiO_2_ ratio | 1.19 [0.92; 1.55] | 0.184 |
| pH | 0.50 [0.15; 1.65] | 0.253 |
| Only NIV | 1.33 [0.83; 2.12] | 0.238 |
| IMV | 1.83 [1.14; 2.97] | 0.013 |
| Limitation of therapeutic effort | 2.91 [2.06; 4.12] | <.001 |
| Respiratory infection as cause of AECOPD | 0.82 [0.62; 1.09] | 0.169 |
|  | | |
| **Timing to ICU admission** | | |
| ICU admission > 24h and ≤ 7 days after hospital admission | 1.54 [1.01; 2.36] | 0. 046 |
| ICU admission > 7 days after hospital admission | 1.42 [0.97; 2.08] |  |
| Direct ICU admission or < 24h after hospital admission | 1 [. ; .] |  |
|  | | |
| **Characteristics of COPD disease** | | |
| Very severe COPD | 0.98 [0.68; 1.42] | 0.669 |
| COPD severity unknown | 1.21 [0.77; 1.89] |  |
| No very severe COPD | 1 [. ; .] |  |
