# Supplementary material for: Corticosteroids for severe acute exacerbations of chronic obstructive pulmonary disease in intensive care: From the French OUTCOMEREA cohort
Source: PLoS One. 2023 Apr 19;18(4):e0284591. doi: 10.1371/journal.pone.0284591 (PMC10115304; doi:10.1371/journal.pone.0284591)
Supplement: S1 File — (DOCX) [file pone.0284591.s014.docx]

**Corticosteroids for severe acute exacerbations of chronic obstructive pulmonary disease in intensive care: from the French OUTCOMEREA cohort. Supporting information.**

**Authors:** Louis-Marie Galerneau; Sébastien Bailly; Nicolas Terzi; Stéphane Ruckly; Maité Garrouste-Orgeas; Yves Cohen; Vivien Hong Tuan Ha; Marc Gainnier; Shidasp Siami; Claire Dupuis; Michael Darmon; Jean-Marie Forel; Florian Sigaud; Christophe Adrie; Dany Goldgran-Toledano; Alexis Ferré; Etienne de Montmollin; Laurent Argaud; Jean Reignier; Jean-Louis Pepin; Jean-François Timsit; on behalf of the OUTCOME REA network

**OutcomeRea^TM^ database**

OutcomeRea^TM^ is an ongoing prospective observational collaborative multicentre database. All treatments and prescribed medications are prospectively collected and entered by senior ICU physicians and clinical research assistants in the participating ICUs every day. All codes and definitions characterizing disease, comorbidities and outcomes were established prior to study initiation and have been previously described.(1) Clinical and outcome data are prospectively included every day in the database for a random sample of patients admitted to 32 French ICUs. Of these 32 participating ICUs (including 18 university hospitals), 16 were polyvalent or surgical ICUs and 16 were primary medical ICUs. All data concerning the studied variables were recorded in the OutcomeRea^TM^ database and missing data were retrieved from medical records during data-cleaning.

**E References**:

E1. Lautrette A, Garrouste-Orgeas M, Bertrand P-M, Goldgran-Toledano D, Jamali S, Laurent V, et al. Respective impact of no escalation of treatment, withholding and withdrawal of life-sustaining treatment on ICU patients’ prognosis: a multicenter study of the Outcomerea Research Group. Intensive Care Med. 2015 Oct;41(10):1763–72.
