# Supplementary material for: Corticosteroids for severe acute exacerbations of chronic obstructive pulmonary disease in intensive care: From the French OUTCOMEREA cohort
Source: PLoS One. 2023 Apr 19;18(4):e0284591. doi: 10.1371/journal.pone.0284591 (PMC10115304; doi:10.1371/journal.pone.0284591)
Supplement: S2 File — (DOCX) [file pone.0284591.s015.docx]

**Precisions on statistical analysis**

As there were few missing values, simple imputations were performed by stratifying on age and sex, except for height, weight, PaO_2_/FiO_2_ ratio and arterial blood pH for which multiple imputation were performed. Twenty imputed datasets were created to consider variables with 20% or less of missing values. Concerning survival at 28 days, patients discharged home before D-28 but whose status was unknown at D-28 were considered alive on D-28.

The small centres (centres with less than 45 admissions for AECOPDs over the study period) were grouped into one single centre for the analysis. When the distribution of variables was not a normal distribution, a logarithmic transformation could be performed for the analysis for BMI and PaO_2_/FiO_2_ratio. The characteristics of the two groups (corticosteroids at admission vs no-corticosteroids at admission) were compared using the Chi-squared test for qualitative data and Mann–Whitney test for quantitative data.

To estimate the average causal effect of corticosteroids at admission on endpoints, an inverse probability of treatment weight estimator (IPTW) estimator was used.

A first step univariable analysis of baseline variables associated with corticosteroids administration at admission and the composite outcome was performed to identify clinically relevant variables and variables associated to the exposition and to the composite outcome (with p values <0.05). Second, after validation of the absence of correlation between predictor variables and of the log-linearity of the variables, these variables were introduced in a non-parsimonious multivariable logistic regression model the IPTW for individual patients. Third, the IPTW was stabilized by multiplying the weights by the probability of observing the events (corticosteroids administration at admission) in the overall population. Fourth, a logistic regression, using the IPTW, was used to assess the impact of corticosteroids at admission on the composite outcome (death or invasive ventilation at D-28 after admission in ICU). For subgroup analyses, the weight model and the IPTW were computed for each subgroup before subgroup analysis and a Cochrane Q test was performed to test the heterogeneity of the results in these subpopulations.

The variables selected to compute individual weights for corticosteroid treatment at admission (IPTW) were: age, sex, BMI, SOFA Day-1, PaO_2_/FiO_2_ ratio at admission in ICU, pH at admission in ICU, NIV as ventilatory support at admission in ICU (24 first hours in ICU), IMV at admission in ICU (24 first hours in ICU), limitation of therapeutic effort at admission in ICU, respiratory infection as cause of AECOPD, severity of COPD disease, year, centre (Weight model used to calculated IPTW in online data supplement). For the double robust estimation, these same variables were kept for multivariable model.

For secondary outcomes, logistic regression, cox models (for survival analysis) or negative binomial regressions (for lengths of stay, ventilator-free days (VFD) and antibiotic-free days (AFD)) using the IPTW was used (details in supplementary data).

Variables used in the IPTW model (clinically relevant variables and variables with p values <0.05) were introduced in the multivariable model to obtain the final multivariable model (doubly robust estimation). Centre was introduced as fixed effects in the multivariable model. Year was introduced as continuous variable in the multivariable model, in order to take into account the effect of year. Indeed, we observed a decrease in the prescription of corticosteroid therapy over years in a previous analysis of our population (submitted for publication), the details of which are presented in this Online Data Supplement.

The comparison of the prevalence of side effects of corticosteroids between the two groups with or without corticosteroids at admission was performed with khi2 Test and Wilcoxon-Mann-Whitney Test
