# Supplementary material for: Corticosteroids for severe acute exacerbations of chronic obstructive pulmonary disease in intensive care: From the French OUTCOMEREA cohort
Source: PLoS One. 2023 Apr 19;18(4):e0284591. doi: 10.1371/journal.pone.0284591 (PMC10115304; doi:10.1371/journal.pone.0284591)
Supplement: S3 File — (DOCX) [file pone.0284591.s016.docx]

**Side effects definitions**

Diagnoses of infectious events such as bacteraemia, pneumonia, catheter infection, urinary tract infection, cholecystitis, tracheobronchitis, viral infection and microbial pathogens were extracted from the database. Infectious events occurring more than 48 hours after hospitalization in the ICU were considered as nosocomial infectious events. Pneumonia occurring after more than 48 hours of invasive mechanical ventilation were considered as ventilator-associated pneumonia.

Maximum systolic blood pressure, creatinine blood levels, urea blood levels, fasting blood glucose levels, potassium blood levels, digestive bleeding events, gastric protective agent prescription and digestive endoscopy were also extracted from the database.

Acute kidney injury during ICU Stay (n=1164) was assessed according to the KDIGO clinical practice guidelines. (E2) Hyperglycaemia was defined by a fasting blood glucose level >11 mmol/L, hypoglycaemia was defined by a fasting blood glucose level <3 mmol/L, hypokalemia was defined by a potassium blood level <3.5 mmol/L.

**E References**:

E2. Khwaja A. KDIGO clinical practice guidelines for acute kidney injury. Nephron Clin Pract. 2012;120(4):c179-184.
