# Supplementary material for: Corticosteroids for severe acute exacerbations of chronic obstructive pulmonary disease in intensive care: From the French OUTCOMEREA cohort
Source: PLoS One. 2023 Apr 19;18(4):e0284591. doi: 10.1371/journal.pone.0284591 (PMC10115304; doi:10.1371/journal.pone.0284591)
Supplement: S4 File — Decrease in the prescription of corticosteroid therapy over the years. (DOCX) [file pone.0284591.s017.docx]

**Statistical analysis. Decrease in the prescription of corticosteroid therapy over the years.**

To evaluate trends over time, we performed an analysis of the evolution of the relevant variables over time using a mixed model taking the time in years into account as a fixed continuous variable; the adjustment variables were fixed variables and the hospital centre was a random effect. This model allowed us to assess the variation of the studied variable over the follow-up period. The small centres (centres with less than 45 admissions for AECOPDs over the study period) were grouped into one single centre for the analysis. Adjustments were performed on age, sex, BMI, the Simplified Acute Physiology Score (SAPS) II, decision for limitation of therapeutic effort and severity of COPD. A 2-sided alpha threshold of .05 was considered significant. Statistical analyses were performed using SAS 9.4 software (SAS Institute, Cary, NC, USA).

We observed a decrease in the prescription of corticosteroids (-4.75±1.18 %/year, p<0.01) and of prescription of corticosteroids > 5 days among patients with ICU length of stay ≥ 7 days (-6.01±2.52 %/year, p=0.02).
