# Supplementary material for: Corticosteroids for severe acute exacerbations of chronic obstructive pulmonary disease in intensive care: From the French OUTCOMEREA cohort
Source: PLoS One. 2023 Apr 19;18(4):e0284591. doi: 10.1371/journal.pone.0284591 (PMC10115304; doi:10.1371/journal.pone.0284591)
Supplement: S5 File — (DOCX) [file pone.0284591.s018.docx]

**OUTCOMEREA NETWORK**

***Scientific Committee*:** Jean-François Timsit (Medical and Infectious Diseases ICU, Bichat-Claude Bernard Hospital, Paris, France; UMR 1137 Inserm –Paris Diderot university IAME, F75018, Paris); Elie Azoulay (Medical ICU, Saint Louis Hospital, Paris, France); Maïté Garrouste-Orgeas (Paliative care, Institut Franco Britanique, Paris, France); Jean-Ralph Zahar (Infection Control Unit, Angers Hospital, Angers, France); Bruno Mourvillier (Medical ICU, CHU Reims,France); Michael Darmon (Medical ICU, APHP Saint Louis hospital Paris, France).

***Biostatistical and Information System Expertise*:** Jean-Francois Timsit (Medical and Infectious Diseases ICU, Bichat-Claude Bernard Hospital, Paris, France; UMR 1137 Inserm –Paris Diderot university IAME, F75018, Paris); Corinne Alberti (Medical Computer Sciences and Biostatistics Department, Robert Debré Hospital, Paris, France); Stephane Ruckly (OUTCOMEREA organization and Inserm UMR 1137 IAME, F75018, Paris); Sébastien Bailly (Grenoble Alpes University, INSERM 1300, HP2, Grenoble, France) and Aurélien Vannieuwenhuyze (Tourcoing, France).

***Investigators of the OUTCOMEREA Database*:** Christophe Adrie (ICU, CH Melun, and Physiology, Cochin Hospital, Paris, France); Carole Agasse (Medical ICU, university hospital Nantes, France); Bernard Allaouchiche (ICU, Hospices civils de Lyon, Lyon sud, Lyon, France); Olivier Andremont (ICU, Bichat Hospital, Paris, France); Pascal Andreu (CHU Dijon, Dijon, France); Laurent Argaud (Medical ICU, Hospices Civils de Lyon, Lyon, France); Claire Ara-Somohano (Medical ICU, University Hospital, Grenoble, France); Elie Azoulay (Medical ICU, Saint Louis Hospital, Paris, France); Francois Barbier (medical-surgical ICU, Orléans, France), Jean-Pierre Bedos (ICU, Versailles Hospital, Versailles, France); Thomas Baudry (Medial ICU, Edouard Heriot hospital, Lyon France), Julien Bohé (ICU, Hôpital Pierre Benite, Lyon France), Lila Bouadma (ICU, Bichat Hospital, Paris, France); Jeremy Bourenne (Réanimation des urgences, Timone-2; APHM, Marseille, France); Noel Brule (medical ICU, university hospital Nantes, France); Frank Chemouni (Grand Hôpital de l’Est Francilien Site Marne La vallée ; Polyvalent ICU, Jossigny Polyvalent ICU) ; Julien Carvelli (Réanimation des urgences, Timone-2; APHM, Marseille, France); Elisabeth Coupez (ICU, G Montpied Hospital, Clermont-Ferrand, France); Martin Cour Medial ICU, Edouard Heriot hospital, Lyon France), Michael Darmon (ICU, APHP St louis, Paris France); Claire Dupuis (ICU, G Montpied Hospital, Clermont-Ferrand, France), Etienne de Montmollin (ICU, Bichat Hospital, Paris, France), Loa Dopeux (ICU, G Montpied Hospital, Clermont-Ferrand, France); Anne-Sylvie Dumenil (Antoine Béclère Hospital, Clamart, France); Claire Dupuis (Bichat hospital and UMR 1137 Inserm –Paris Diderot university IAME, F75018, Paris, France), Jean-Marc Forel (AP HM, Medical ICU, Hôpital Nord Marseille), Marc Gainnier (Réanimation des urgences, Timone-2; APHM, Marseille, France), Charlotte Garret (Medical ICU, university hospital Nantes, France); Dany Goldgran-Tonedano ( CH le Raincy-Montfermeil; France); Steven Grangé (ICU, CHU Rouen, France), Antoine Gros (ICU, Versailles Hospital, Versailles, France), Hédia Hammed (CH le Raincy-Montfermeil) ; Akim Haouache (Surgical ICU, H Mondor Hospital, Créteil, France); Tarik Hissem (ICU, Eaubonne, France), Vivien Hong Tuan Ha (ICU, CH Meaux, France); Sébastien Jochmans (ICU, CH Melun); Jean-Baptiste Joffredo (ICU, G Montpied Hospital, Clermont-Ferrand, France); Hatem Kallel (ICU, Cayenne General Hospital, Cayenne, France); Guillaume Lacave (ICU, Versailles Hospital, Versailles, France), Virgine Laurent (ICU, Versailles Hospital, Versailles, France), Alexandre Lautrette (ICU, G Montpied Hospital, Clermont-Ferrand, France); Clément Le bihan (ICU, Bichat Hospital, Paris, France), Virgine Lemiale (Medical ICU, Saint Louis Hospital, Paris, France); David Luis (Médecine intensive et réanimation, CH Simone Veil, Beauvais, France), Guillaume Marcotte (Surgical ICU, Hospices Civils de Lyon, Lyon, France); Jordane Lebut (ICU, Bichat Hospital, Paris, France); Bruno Mourvillier (ICU, Bichat Hospital, Paris, France); Benoît Misset (ICU, Saint-Joseph Hospital, Paris, France); Bruno Mourvillier (ICU, Medical ICU, Reims France); Mathild Neuville (ICU, Foch Hospital, Paris, France) ; Laurent Nicolet (Medical ICU, university hospital Nantes, France); Johanna Oziel (Medico-surgical ICU, hôpital Avicenne APHP, Bobigny, France), Laurent Papazian (Hôpital Nord, Marseille, France), Juliette Patrier (ICU, Bichat Hospital, Paris, France), Benjamin Planquette (pulmonology ICU, George Pompidou hospital Hospital, Paris, France); Aguila Radjou (ICU, Bichat Hospital, Paris, France), Marie Simon (Medial ICU, Edouard Heriot hospital, Lyon France), Romain Sonneville (ICU, Bichat Hospital, Paris, France), Jean Reignier (Medical ICU, university hospital Nantes, France); Bertrand Souweine (ICU, G Montpied Hospital, Clermont-Ferrand, France); Carole Schwebel (ICU, A Michallon Hospital, Grenoble, France); Shidasp Siami (ICU, Eaubonne, France); Romain Sonneville (ICU, Bichat Hospital, Paris, France); Nicolas Terzi (ICU, A Michallon Hospital, Grenoble, France ) ; Gilles Troché (ICU, Versailles Hospital, Versailles, France); Fabrice Thiollieres (ICU, Hospices civils de lyon, Lyon sud, Lyon, France) ; Guillaume Thierry (ICU, St Etienne, France); Guillaume Van Der Meersch (Medical Surgical ICU, university hospital Avicenne), Marion Venot (Medical ICU, Saint Louis Hospital, Paris, France); Florent Wallet (ICU, Hospices civils de lyon, Lyon sud, Lyon, France) : Sondes Yaacoubi (CH le Raincy-Montfermeil); Olivier Zambon (medical ICU, university hospital Nantes, France); Jonathan Zarka (Réanimation polyvalente, centre hospitalier de Marne la Vallee, France).

***Study Monitors****: Mireille Adda, Vanessa Vindrieux, Marion Provent, Sylvie de la Salle, Pauline Enguerrand, Vincent Gobert, Stéphane Guessens, Helene Merle, Nadira Kaddour, Boris Berthe, Samir Bekkhouche, Kaouttar Mellouk, Mélaine Lebrazic, Carole Ouisse, Diane Maugars, Christelle Aparicio, Igor Theodose, Manal Nouacer, Veronique Deiler, Fariza Lamara, Myriam Moussa, Atika Mouaci, Nassima Viguier.*
